# Offline Evaluation of Recommender Systems in a User Interface With Multiple Carousels

**DOI:** 10.3389/fdata.2022.910030

**Published:** 2022-06-09

**Authors:** Maurizio Ferrari Dacrema, Nicolò Felicioni, Paolo Cremonesi

**Affiliations:** ^1^Department of Electronics Information and Bioengineering, Politecnico di Milano, Milano, Italy; ^2^ContentWise, Milano, Italy

**Keywords:** recommender systems, user interface, evaluation, layout optimization, industrial scenario, carousel interface

## Abstract

Many video-on-demand and music streaming services provide the user with a page consisting of several recommendation lists, i.e., *widgets* or *swipeable carousels*, each built with specific criteria (e.g., most recent, TV series, etc.). Finding efficient strategies to select which carousels to display is an active research topic of great industrial interest. In this setting, the overall quality of the recommendations of a new algorithm cannot be assessed by measuring solely its individual recommendation quality. Rather, it should be evaluated in a context where other recommendation lists are already available, to account for how they complement each other. The traditional offline evaluation protocol however does not take this into account. To address this limitation, we propose an *offline evaluation protocol for a carousel setting* in which the recommendation quality of a model is measured by how much it improves upon that of an already available set of carousels. We also propose to extend ranking metrics to the two-dimensional carousel setting in order to account for a known position bias, i.e., users will not explore the lists sequentially, but rather concentrate on the top-left corner of the screen. Finally, we describe and evaluate two strategies for the ranking of carousels in a scenario where the technique used to generate the two-dimensional layout is agnostic on the algorithms used to generate each carousel. We report experiments on publicly available datasets in the movie domain to show how the relative effectiveness of several recommendation models compares. Our results indicate that under a carousel setting the ranking of the algorithms changes sometimes significantly. Furthermore, when selecting the optimal carousel layout accounting for the two dimensional layout of the user interface leads to very different selections.

## 1. Introduction

The general goal of a recommendation system is to help the users navigate a large number of options at their disposal by suggesting a limited number of relevant results. Traditionally, the focus of newly developed recommendation systems is to generate the best possible ranked list of results, refer to (Herlocker et al., 2004; Cremonesi et al., 2010; Sanderson and Croft, 2012). A common assumption in almost all research works is that the recommendations will be provided to the user as a single list which will be explored in its order from the first element to the last.

**Figure 1:**
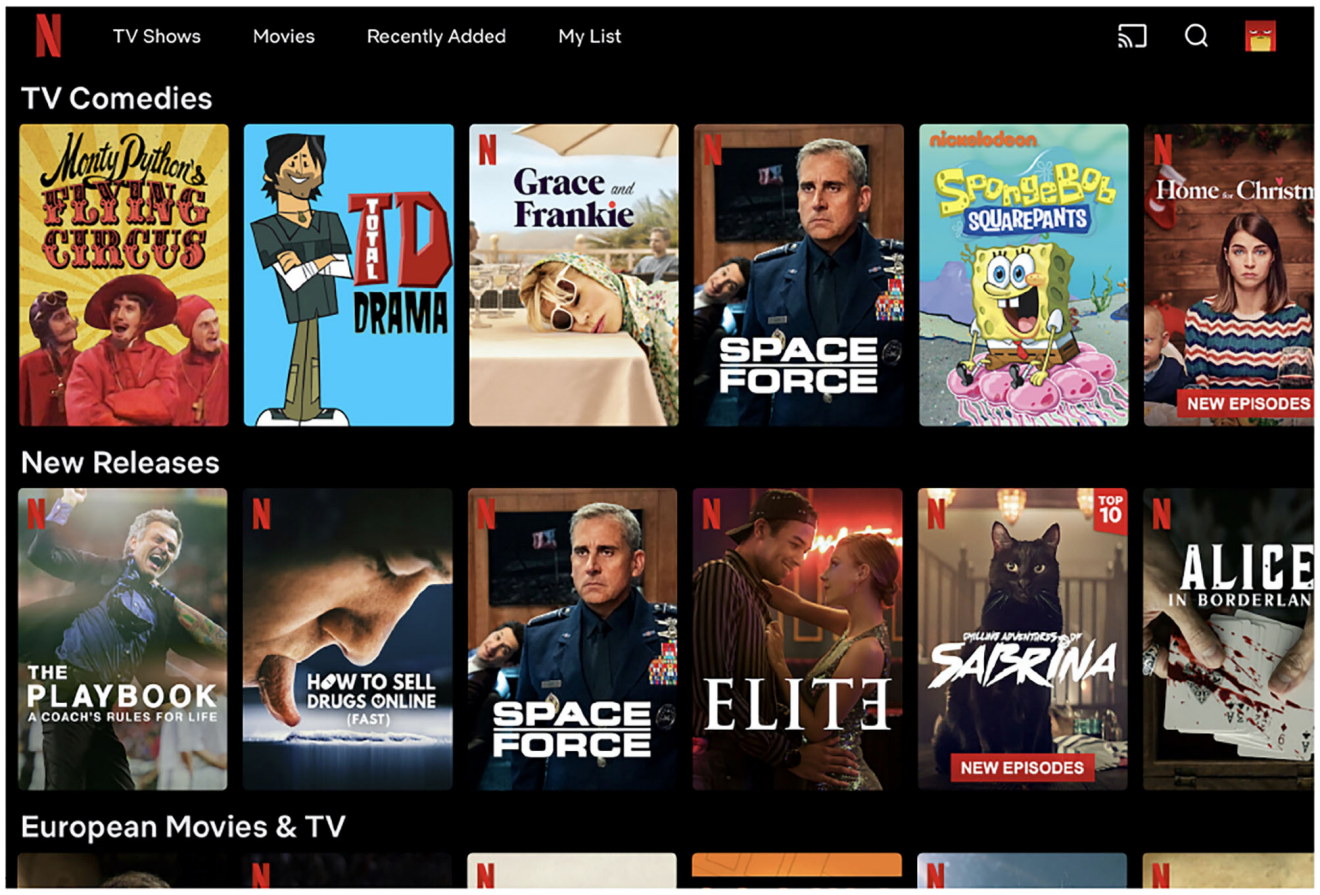
The Netflix homepage, an example of the carousel user interface in the multimedia streaming domain.

However, many industrial applications provide users with a two-dimensional layout of recommendations. Examples are video on demand (e.g., Netflix, Amazon Prime Video) and music streaming services (e.g., Spotify). In these scenarios the user is provided with an interface composed of multiple rows, each row containing thematically consistent recommendations, e.g., most recent, most popular, editorially curated (see Figure 1), see (Wu et al., 2016; Gruson et al., 2019; Bendada et al., 2020; Elahi and Chandrashekar, 2020; Pérez Maurera et al., 2020). These rows are referred to as *widgets, shelves*, or *carousels*. In a carousel interface scenario, the user satisfaction depends both on the entire set of carousels shown to the user, rather than on a single list, and their relative positions. Although finding appropriate combinations of algorithms and ranking them to provide the user with a personalized page is an active research topic of significant industrial interest (Wu et al., 2016; Ding et al., 2019; Bendada et al., 2020), the amount of work done on the design and evaluation of recommender systems based on multiple carousels is limited to few articles based on proprietary datasets.

The two-dimensional layout is becoming more and more common due to *content aggregators*, i.e., services or platforms that aggregate within a single UI the content (and recommendations) provided by different services, e.g., Android TV, Sky Q, Prime Video. Although two dimensional layouts are not new, their semantics have changed over time. The first two-dimensional interfaces used a grid layout which differs from the currently used carousel layout because the rows did not have clearly identifiable differences in their semantics. The first to introduce the carousel interface was Netflix, mainly because the carousel interface allows to label the recommendation lists in a way that is much more explainable, e.g., a user that has seen the movie Dune may be provided with a carousel labeled “Movies similar to Dune”. The carousel layout has several characteristics that distinguish it from the traditional single-list scenario and other grid layouts. Three important factors that should be taken into account are: (*i*) different carousels are generated by different and independent machine learning pipelines; (*ii*) some carousels are editorially curated and cannot be modified, business constraints may play a role in what contents should be promoted; (*iii*) content aggregators show carousels that are generated by different content providers and the content aggregator can personalize the layout but has no control on the recommendation lists content.

**Figure 2:**
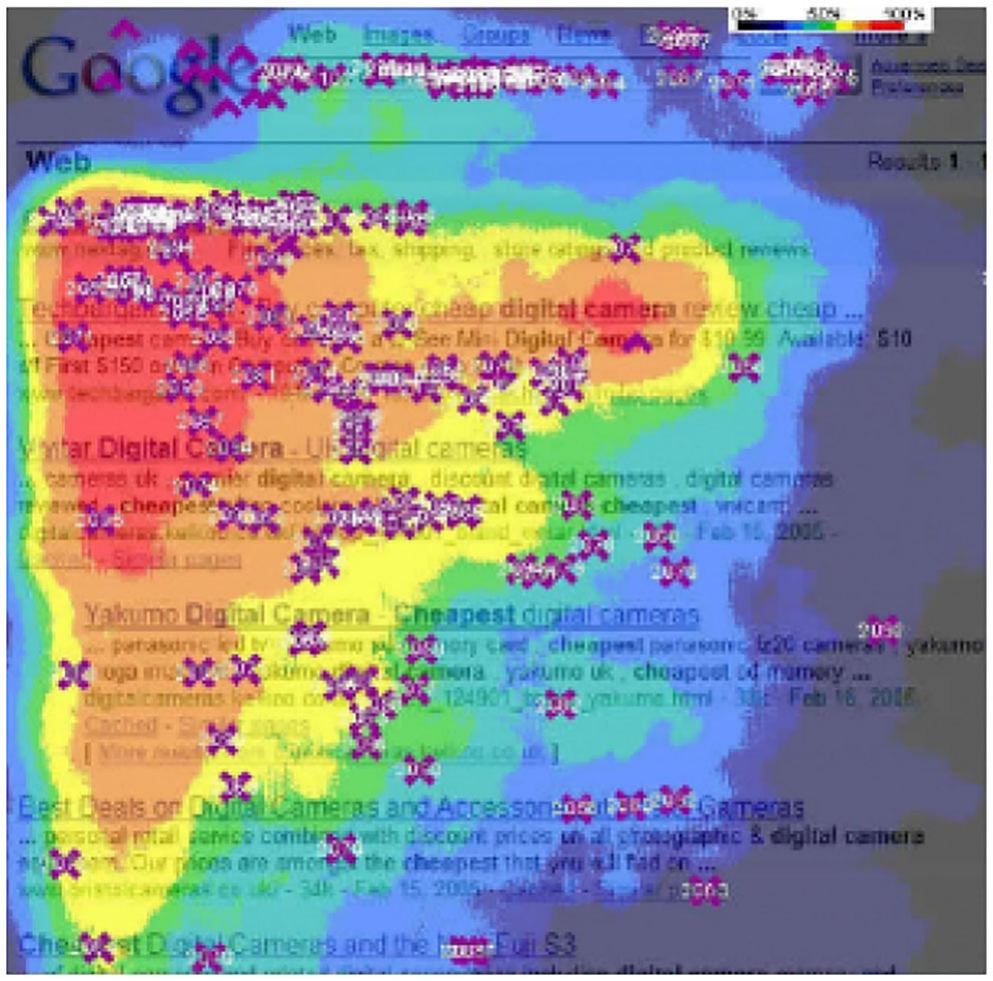
Heatmap from real usage data of how users navigate a simple search result page, the attention is focused on a top-left *golden triangle* and progressively decreases moving to the right and the bottom (Chierichetti et al., 2011).

Among the challenges researchers face is the absence of a standardized evaluation procedure that accounts for these factors and for how the users navigate a two dimensional interface. One of the strategies that are adopted to evaluate offline in a carousel setting is to create a single recommendation list that concatenates all carousels provided to the user (Gruson et al., 2019). This strategy is, however, not realistic as it assumes that all recommendation lists will be centrally collected and processed (e.g., removing duplicates) and that the user explores the two-dimensional user interface sequentially, both of which are not realistic assumptions. In reality, due to strict real-time requirements or business constraints, the recommendation lists cannot be collected and modified in a centralized post-processing step. Furthermore, users tend to start from the top-left corner of the screen and then proceed to explore the items both to the right and to the bottom (Kammerer and Gerjets, 2010; Zhao et al., 2016). This behavior has been long known and is referred to as the “golden triangle” or “F-pattern.” An example taken from an information retrieval application (Chierichetti et al., 2011) is shown in Figure 2.

In this article, we address the problem of evaluating recommender systems in which the user interface is composed of multiple and independent carousels. This problem is referred to as the page personalization problem. This study extends prior studies by Felicioni et al. (2021b) and Felicioni et al. (2021a) and has the following contributions:

- The characteristics of a carousel interface are defined in detail as well as the relevant tasks;
- An evaluation procedure is proposed that accounts for the characteristics of the carousel interface and adapts commonly used accuracy, ranking, and beyond-accuracy metrics;
- An extension of the widely used NDCG ranking metric is proposed to account for how users navigate a two dimensional user interface;
- Two simple greedy strategies are proposed as baselines for the task of carousel ranking; the strategies are agnostic on how each carousel has been created;
- The experimental analysis shows the impact of accounting for the carousel interface and two dimensional layout, yielding to sometimes very different results compared to traditional single-list evaluation approaches.

## 2. Related Study

Most of the studies targeting a carousel user interface come from industrial research with evaluation performed through A/B testing. This demonstrates the importance, at the industrial level, of identifying an adequate combination of carousels to show to the user. However, it may also indicate that the scarcity of appropriate datasets and the lack of a standardized offline evaluation protocol is hampering researchers who do not have easy access to online evaluation infrastructure, preventing them to work on the topic.

Wu et al. (2016) analyze the problem of optimizing the position of the carousels on the interface of Netflix, a popular online video streaming service. The authors propose a model based on the notion that the benefit of recommending a certain item depends on how novel it is relative to the previous recommendations, a concept referred to as *submodularity*. Their algorithm is able to leverage scrolls and navigation feedback to dynamically optimize the user interface. The evaluation is done both online and offline. For the offline evaluation standard metrics are reported (MRR and Precision) considering a carousel as a single item that is relevant if it contains at least a relevant recommendation, therefore, not accounting for the ranking within the carousel. Gruson et al. (2019) optimize the homepage of Spotify, an online music streaming service, which recommends playlists in thematic rows referred to as *shelves*. The article evaluates a series of policies to rank the most relevant playlists for a user. The policies are ranked according to first an online evaluation and then an offline evaluation. The two rankings are then compared and de-biasing techniques are discussed to improve the correspondence of offline evaluations with online ones. In the offline evaluation, the carousels are sequentially concatenated as a single long recommendation list. Bendada et al. (2020) propose a contextual multi-armed bandit online approach to optimize the two-dimensional layout of the homepage of the online music streaming service Deezer. Each user is shown a set of carousels which the user can swipe to reveal further items. The system does not log the swipe actions. To estimate swipe actions the authors rely on the *cascade model* (Craswell et al., 2008) assuming the user has seen all items before the one they interacted with and therefore has swiped and ignored them. The policies are evaluated both online and offline with expected cumulative regrets. Ding et al. (2019) target the problem of whole page optimization for the homepage of Amazon Video, a video streaming service. They assume that a set of widgets is already available and that the objective is to select which widgets to show and in which order while also accounting for the business constraints of the homepage. The task of carousel selection was also tackled by Ferrari Dacrema et al. (2021) who proposed a quadratic optimization problem to decide which carousels should be selected among a pool of available ones. The optimization problem was proposed in a formulation that allowed it to be solvable using quantum computing technologies. Felicioni et al. (2021a) proposed an extension to NDCG that accounts for the two-dimensional structure of the user interface with a different ranking discount term.

To the best of our knowledge, Elahi and Chandrashekar (2020) is the only work that tries to account for the two-dimensional structure of the page during the algorithm training phase. However, this scenario is unrealistic in almost all industrial settings, as different carousels are generated independently, and the service that manages the layout of the page has no control over the content of each carousel.

It can be seen from these studies that the carousel scenario is not yet approached in a homogeneous way and that different articles account for different factors and evaluate them in different ways. Still, some aspects of the carousel scenario emerge such as the need to account for how carousels complement each other (i.e., avoid duplicate recommendations), the desire to minimize the user actions required, the tasks of carousel selection and ranking, and the lack of a common evaluation procedure.

## 3. Characteristics of a Carousel Interface

The carousel interface layout and the way it is usually generated by video-on-demand and music streaming platforms have important characteristics that distinguish it from a single-list setup, refer to Felicioni et al. (2021a). While a carousel layout may seem similar to a traditional merge-list ensemble, where multiple recommendation lists are combined into one, this is not the case. In a real scenario, multiple constraints play a role and must be taken into account: (*i*) the two dimensional structure of the layout, (*ii*) how the recommendation lists are created, and (*iii*) how the users interact with such interfaces.

- **Layout structure:** The two dimensional user interface of almost all devices (TV sets, personal computers, tablets) is organized with multiple horizontal carousels, where each carousel is generated according to a certain (often explainable) criteria e.g., most recent, most popular, because you watched, editorially curated (refer to Figure 1). Some carousels or recommendations may be hidden due to limited page size and be accessible only via user actions (i.e., vertical and horizontal swipe).
- **Recommendation lists:** The lists shown to the users within each carousel are generated with different algorithms or by different providers and independently from each other (i.e., each algorithm or provider is not aware of the existence of the other lists or their content). Consider, e.g., *content aggregators*, which combine carousels from different providers, e.g., Sky, Youtube, Netflix, Prime Video, etc. No single post-processing step is applied, e.g., to remove items duplicated across different carousels. This is due to various reasons: (*i*) it is difficult to perform further processing under the strict real-time requirements of a recommendation system; (*ii*) the business constraints a content aggregator is subject to may prevent changes to the recommendation lists that are generated by the providers; (*iii*) some carousels are manually curated and contain a fixed recommendation list. Due to this, while each individual recommendation list does not contain duplicates, the same item may be recommended in multiple carousels. The Netflix homepage shown in Figure 1 contains the TV series *Space Force* both in the *TV Comedies* and *New Releases* carousels, which is an example of a case where duplicates are not removed.
- **User behavior:** The users will focus on the top-left triangle of the screen rather than exploring the carousels sequentially. This is usually called the *golden triangle*, refer to Figure 2. Furthermore, they will explore the recommendations in different ways according to which actions they need to perform in order to reveal them. Usually, users tend to navigate more easily with simple swipes rather than repeated mouse clicks, hence their behavior, as it is known, will change according to the device (e.g., personal computer, smartphone, tablet, Smart TV).

### 3.1. Tasks

Within the context of a carousel layout, two orders of tasks are relevant, one is the traditional development of effective recommendation algorithms that can be used to populate a single carousel, while the second is to create the two-dimensional interface layout by deciding which carousels should be displayed and where. While developing new and more effective recommendation algorithms has been a core research topic for two decades, much less attention has been paid to the second problem. Therefore, this article focuses in particular on how to create the carousel layout in a scenario where the technique used to generate the layout is agnostic to how the recommendation lists are created. Three sub-tasks of particular importance can be defined:

- **Carousel selection:** given a set of recommendation lists, the task is to decide which subset of them to choose. As an example consider a scenario where we want to provide the user with a carousel related to sports, and the goal is to decide *which* sports to include based on the user's preferences. Similarly, there may be recommendation lists for new releases of specific genres, e.g., comedies, fantasy, science fiction, drama, etc, and again the goal is to decide which ones to show. A crucial challenge of this task is that it requires taking into account how the recommendation lists complement each other Wu et al. (2016). Clearly, the aim is to ensure the user is provided with the best possible set of recommendations, but it is not beneficial to provide the user with the same recommendations in multiple carousels. Due to this, it is not sufficient to simply select the recommendation lists that have the best accuracy when measured independently because they may be redundant. Furthermore, different users or different groups of users may have different optimal layouts. Certain constraints may apply to the types of carousels, e.g., one may wish that there should always be a popularity-based non personalized algorithm, an editorially curated one, a genre-specific one, and a further personalized model.
- **Carousel ranking:** Given an already defined set of carousels, the task is to decide in which order to display them. A carousel ranking task is equivalent to starting from a default ranking, e.g., editorially curated and developing a re-ranking strategy. The goal is to choose the ordering that allows the user to rapidly see the most relevant recommendations. In a similar way as recommendations are ranked within an individual list, in a two dimensional layout carousels should be ranked according to both (i) the user navigation pattern as it emerges from the golden triangle and (ii) the user actions required to access certain positions. The challenge of this task is to account for how the user navigates a two dimensional user interface as well as what portion of the recommendation lists are immediately visible to the user and which require user actions. Due to this, ordering the carousels according to their decreasing recommendation quality will lead to suboptimal layouts. Furthermore, business constraints may apply to the relative ordering of some of the carousels.
- **Carousel insertion:** Given an already available carousel interface, the task is to decide where a new carousel should be added without altering the relative position of the available ones. As opposed to carousel selection and ranking, the insertion of a carousel is an incremental task and does not require to search for an optimal layout anew. As an example, consider a content aggregator that displays recommendations provided by several video on demand services the user has subscribed to. If the user subscribes to a new service, we want to include that new carousel in the already existing layout, in a position that maximizes the user satisfaction within possible business constraints.

All the described tasks assume that the recommendation lists that can be used as carousels are already available and the goal is to decide what is the optimal layout. This requires developing an evaluation procedure that is tailored to the specific nature of the carousel setting as well as extending traditional evaluation metrics, refer to Section 4.

As a last note, while this article considers the creation of the carousel layout given that (i) all carousels are already available and (ii) the layout manager is not aware of the algorithms used to fill the carousels, it is indeed possible to combine the carousel ranking tasks at the page level with the item ranking task within each carousel.

## 4. Evaluation Procedure for a Carousel Interface

This section describes an evaluation procedure tailored for a carousel user interface, which accounts for its characteristics and the constraints that are normally present in a real industrial setting. While the traditional evaluation assesses the recommendation quality of a single recommendation model, in a carousel scenario the goal of the evaluation is to assess the recommendation quality of a certain layout composed of recommendation lists (either fixed or generated by a specific recommendation model). Once it is possible to evaluate the overall recommendation quality of a single layout it is possible to compare different layouts in order to select the best one. For example, one may wish to select the optimal carousel ranking or to choose which recommendation model should generate a specific carousel. A summary of the notation used is reported in Table 1.

**Figure 3:**
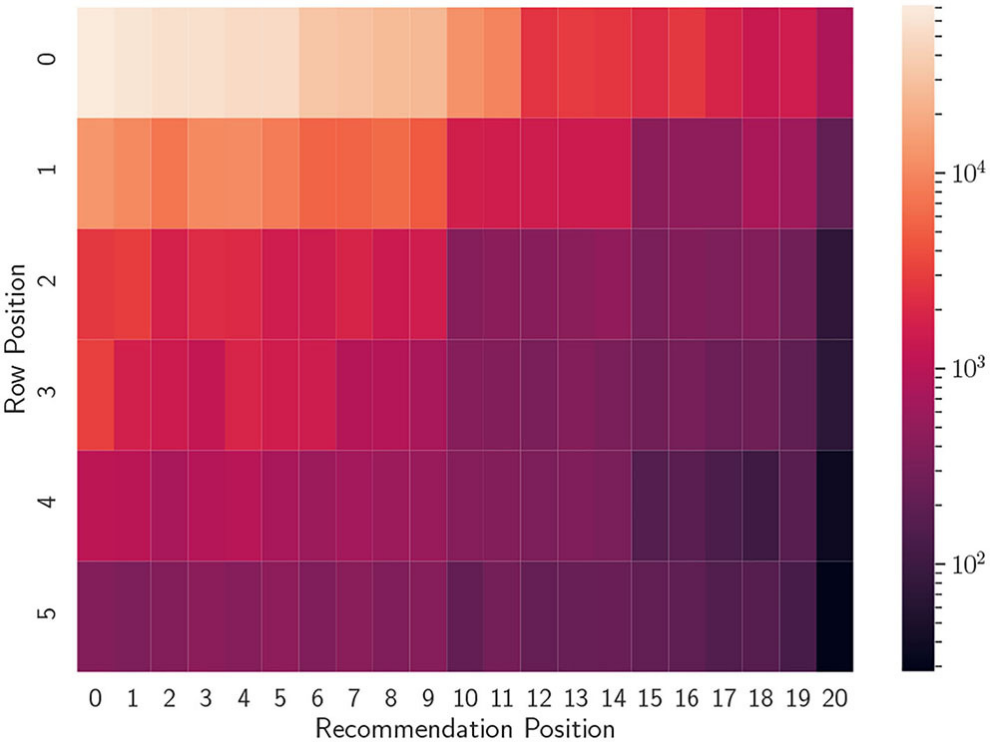
Heatmap of the number of interactions per position on the screen, taken from the dataset presented in Pérez Maurera et al. (2020).

- **Scenario:** The carousel evaluation setting is closely tied to a specific user interface commonly adopted for video on demand and music streaming services. As such, while it could be applied for any domain, we recommend its use be limited to those that employ such carousel interface. Researchers have ample freedom to decide how to generate the carousels (editorially curated lists, recommendation models, etc.).
- **Optimization:** If some of the carousels are generated with recommendation models, the first step is to ensure that all models are adequately optimized. In a traditional offline evaluation, each model is optimized independently by selecting the hyperparameters that optimize its recommendation quality on a validation set (Ferrari Dacrema et al., 2019). The same holds for a carousel scenario. In a carousel setting the recommendation lists provided to the user may come from different sources, e.g., a third party providing the recommendation engine for some carousels, while the platform provides others, Pérez Maurera et al. (2020), the carousels shown to the user may change dynamically between sessions and different users may see a different carousel selection. This means that during the optimization of the models the layout of the user interface is, in general, not known and cannot be used during the optimization phase. For this reason, we recommend all models should be optimized independently and, if assumptions are made on the composition of the carousels, those should be stated clearly. For example, it may be assumed that a popularity-based carousel is present and therefore train the recommendation models specifically to improve the recommendation quality on less popular items.
- **Recommendation:** The recommendations that will be shown to the users are the sequence of all the recommendation lists in the layout. Which lists to include and in which order is part of the experimental setting and will be chosen according to the scenario of interest. All lists have the same length, *H*, corresponding to the horizontal dimension of the interface (i.e., number of columns). Given *V* the number of recommendation lists that are displayed (i.e., the number of rows) the user will receive *l* = *HV* recommendations. Usually, the recommendations come from different providers, and there will be no centralized postprocessing done on the set of all recommendation lists. While a carousel will only contain a certain item at most once, there could be items appearing in more than one carousel.
- **Evaluation:** In a carousel setting the recommendations provided to the user will be displayed with a two-dimensional pattern. A frequent simplification is to concatenate all *V* recommendation lists in a single one of length *l* = *HV* and remove duplicate recommendations. While this allows using traditional metrics (e.g., NDCG, MAP), it makes assumptions that are not consistent with a carousel layout: (*i*) the user explores the lists sequentially; (*ii*) the recommendation lists are centrally collected and postprocessed. In reality, the user will not explore a list sequentially, but rather start from the top left corner and will move in both directions exploring multiple carousels (refer to Figure 3). There may also be items that appear on more than one carousel. When this happens, we must ensure that a correct recommendation is only counted once and with the correct ranking, despite having appeared multiple times in different positions. The correct recommendation should be counted where the user would *see it first*, according to the user's navigation pattern. A detailed description of how the evaluation metrics should be computed is provided in Section 4.1.

**Table 1:** Summary of the notation used for the carousel interface.

**Symbol**	**Description**
*H*	Horizontal dimension of the interface, i.e., number of columns. Corresponds to the length of each recommendation list.
*V*	Vertical dimension of the interface, i.e., number of rows. Corresponds to the number of carousels.
*M*	Total number of recommendation lists that can be used for the carousel layout, *M* ≥ *V*.
*l*	Total number of recommendations provided to the user. When evaluating a single list *l* = *H*. When evaluating a carousel layout *l* = *HV*.
*V*_*h*_	Number of columns of the interface that are immediately visible to the user, *V*_*h*_ ≤ *H*
*V*_*v*_	Number of rows of the interface that are immediately visible to the user, *V*_*v*_ ≤ *V*
δ_*h*_	Number of columns that are revealed after a user action, δ_*h*_ ≤ *V*_*h*_
δ_*v*_	Number of rows that are revealed after a user action, δ_*v*_ ≤ *V*_*v*_

### 4.1. Evaluation Metrics

Evaluation in a carousel setting presents broad similarities with a traditional top-n recommendation scenario. An important difference is the presence of duplicates in the recommendation list and the two-dimensional way users explore the interface, which impacts how ranking metrics may consider the item position. This section describes how accuracy, ranking, and beyond-accuracy metrics should be adapted to a carousel evaluation.

#### 4.1.1. Accuracy Metrics

Accuracy metrics that are based only on the total number of correct recommendations, e.g., Precision, Recall, Hit Rate, etc. can be computed in the same way as in single-list evaluation, provided that each correct recommendation is only counted once even if it appears in multiple carousels.

#### 4.1.2. Ranking Metrics

Adapting ranking metrics to a carousel evaluation requires accounting for the two-dimensional user exploration of the interface at two stages: (*i*) how the *ranking discount* is computed; (*ii*) how correct recommendations appearing in more than one carousel are considered. For traditional top-n ranking with a single recommendation list (or a merge-list embedding), duplicates are removed from the list; therefore, we assume that each recommendation list does not contain duplicates. However, in a carousel evaluation duplicates might occur between carousels. Such duplicates must not be removed in order to maintain the original positions of the recommendations and mimic the real behavior of carousel-based user interfaces. In a carousel evaluation scenario, an item in the recommendation list is relevant, i.e., a correct recommendation, if it meets two conditions:

- The item appears in that user's ground truth;
- The item has been recommended only once, or if it has duplicates, it is the one corresponding to the *best ranking*, i.e., the position where the user is likely to see it first. The question, therefore, becomes defining a new ranking discount that accounts for the user behavior in a carousel interface. Section 5 discusses different discount functions that are applied to NDCG but can be applied seamlessly to other metrics such as MRR.

#### 4.1.3. Beyond-Accuracy Metrics

Another important dimension to be taken into account when evaluating recommendation models is their diversity. There are three ways to measure the diversity of recommendations: (*i*) *individual* or within-list, that measure how diverse are the recommendations received by a single user, e.g., based on the item features or the item popularity in the training data; (*ii*) *list-based*, that consider the diversity of the recommendations received by couples of users; (*iii*) *aggregate* or distributional, that measure how unbalanced is the overall distribution of recommended items at a system level.

In a carousel evaluation setting, most beyond-accuracy metrics can be used seamlessly and computed on the recommendations of all carousels concatenated in a single list and counting the occurrences of duplicate items as well. This is because repetitions of the same items indicate the recommendations have lower diversity. It is possible to calculate correctly several commonly used within-list beyond-accuracy metrics. Examples are Average Popularity, which is based on the average popularity of the recommended items, and Novelty or mean self-information, which is a function of the item popularity in the training data (Zhou et al., 2010). The same applies to distributional diversity such as Item Coverage, which measures the quota of items that have been recommended at least once, as well as to other metrics that that are computed on the global number of times each item has been recommended (the more unbalanced the distribution the less diverse the recommendations are) Shannon diversity, Gini diversity, and Herfindahl Index (Adomavicius and Kwon, 2012; Ferrari Dacrema, 2021).

As opposed to the previous two categories, list-based diversity metrics such as Mean inter-list diversity (MIL) (Zhou et al., 2010) and the equivalent Hamming diversity behave differently. MIL measures the average quota of the recommendation lists any couple of users have in common. Computing MIL with this approach is however very computationally expensive as it is equivalent to computing a user-based KNN similarity model. The original formulation of MIL is developed for the traditional single-list recommendation setting, in which all users receive the same number of recommendations and the recommendations do not contain duplicates. In this scenario, a recent proof by Ferrari Dacrema (2021) shows that MIL is equivalent to the Herfindahl Index and, therefore, is a distributional diversity metric, this means it can be computed in negligible time based on the global number of times each item has been recommended. In a carousel setting, due to the presence of duplicates, the quota of recommendations two users have in common becomes asymmetric. As an example, consider two users *A* and *B* that received 10 recommendations. If they have one item in common this means that 10% of the recommendations are equal. If the common item appears twice in the recommendations received by user *A*, the similarity becomes asymmetric with *A* having 20% of the recommendation list in common with *B*, while *B* having 10% of the list in common with *A*. Removing the duplicates would produce erroneous results since MIL is defined as an arithmetic mean and requires recommendation lists of the same length. Due to this, the equivalence shown in Ferrari Dacrema (2021) does not hold in a carousel setting and it is an open research question whether the effect of the compositions of the specific user recommendation lists would cause MIL to still behave as a distributional diversity metric or not.

In a carousel scenario, it would also be possible to define additional beyond-accuracy metrics that measure the diversity *between carousels*, e.g., measure the overlap between recommendations contained in different carousels shown to a single user. Defining new metrics for this scenario is, however, an open research question that goes beyond the scope of this article.

## 5. Extending One-Dimensional NDCG

The *Discounted Cumulated Gain* (DCG), as well as its *Normalized* version (NDCG) (Järvelin and Kekäläinen, 2000, 2002), are among the most used metrics for the evaluation of ranked lists. These metrics come from the information retrieval domain and are widely used to evaluate recommendation systems. The DCG metric relies on two assumptions:

1. highly relevant results are more valuable for a user;
2. within a list of results, it is preferable to have relevant results in the first positions.

Let *l* be the recommendation list length, i.e., cutoff, *u* a user within the set of existing users *U*, and *j* the position in the recommendation list (note that *j* does not refer to a specific item but rather to a position in the list). The DCG for user *u* is defined as the following discounted sum of gains:

(1) $$\begin{array}{r} DCG_{u}=\overset{l}{\underset{j=1}{\sum }}g_{uj}d_{j} \end{array}$$

The gain function *g*_*uj*_ is responsible for rewarding highly relevant items in position *j* for user *u*, while the discount function *d*_*j*_ introduces a penalization that should increase as position *j* moves toward the end of the list.

Given *r*_*uj*_ as the relevance (e.g., rating) of the item in position *j* of the recommendation list for user *u*, the most used formulations for gain and discount are $g_{uj}=2^{r_{uj}}-1$ and $d_{j}=\frac{1}{\log_{2}\left(j+1\right)}$. Hence, DCG is computed as Burges et al. (2005):

(2) $$\begin{array}{r} DCG_{u}=\overset{l}{\underset{j=1}{\sum }}\frac{2^{r_{uj}}-1}{\log_{2}\left(j+1\right)} \end{array}$$

The DCG for each user is normalized by computing the ideal DCG for that same user, denoted as IDCG_*u*_. While the DCG considers all items in the recommendation list, the IDCG is computed assigning to each item its true relevance (i.e., the one in the test data) and, therefore, obtaining the best possible ranking given that user's ground truth within the available recommendation list of length *l*. The NDCG for user *u* is computed as:

(3) $$\begin{array}{r} NDCG_{u}=\frac{DCG_{u}}{IDCG_{u}} \end{array}$$

Finally, the global NDCG is computed as the average of the NDCG of each user:

(4) $$\begin{array}{r} NDCG=\frac{1}{\left|U\right|}\underset{u\in U}{\sum }NDCG_{u} \end{array}$$

Notice that this formulation is only one of many possible formulations for the DCG. Several other ways of rewarding and discounting results have been proposed in previous research (Kanoulas and Aslam, 2009; Zhou et al., 2014). In the following, we will start from this widely used formulation and extend it. Other types of gain and discount functions can be extended in an analogous way. We leave the analysis of different gains and discounts as future study.

Extending the DCG definition to a carousel setting requires taking into account both the way a user explores a two dimensional interface, following the golden triangle and that only a portion of the recommendations will be immediately visible to the user and further portions will become visible following user actions, e.g., click, swipe. Furthermore, the shape and size of the user interface as well as the user behavior will change according to the device.

In a two-dimensional scenario, the standard DCG definition can be naively adapted in the following way. Let *H* be the horizontal dimension of the interface (i.e., the length of each carousel) and *V* the vertical dimension of the interface (i.e., the number of carousels). The carousels can be concatenated in a single list of length *l* = *V* · *H* items on which the standard DCG formulation can be applied. This strategy assumes that the users will explore all carousels sequentially, from the first to the last, which, as previously discussed, is not consistent with the user behavior and does not account for the interface navigation constraints. Therefore, we suggest researchers *do not* apply this strategy as it does not represent a realistic scenario.

Thus, inspired by Järvelin and Kekäläinen (2000), we make the following assumptions that the two-dimensional DCG should meet:

1. highly relevant results are more valuable for a user;
2. a relevant result is valuable to the user only when it is first seen;
3. within a grid of results, it is preferable to have relevant results close to the top-left corner;
4. it is preferable that relevant items are immediately visible to the user or can be made visible with the least effort, e.g., the least number of user actions, given that actions of different types may require different efforts.

In order to account for this set of assumptions, we propose to extend the DCG metric as a two-dimensional DCG, 2*DCG*, in the following way:

(5) $$\begin{array}{r} 2DCG_{u}=\overset{V}{\underset{j=1}{\sum }}\overset{H}{\underset{k=1}{\sum }}g_{ujk}d_{jk} \end{array}$$

Where *j* ∈ [1, *V*] and *k* ∈ [1, *H*] represent the position within the two-dimensional carousel interface. As in the one-dimensional version, the gain function is responsible for rewarding highly relevant results, according to assumptions (1) and (2). The discount function, instead, should account for the penalty related to the position and number of user actions, according to assumptions (3) and (4).

Inspired by the one-dimensional version, we define $g_{ujk}=2^{r_{ujk}}-1$ where *r*_*ujk*_ is the relevance of item in carousel *j*, position *k*, for user *u*. The discount term will depend on the position in the layout, allowing ample freedom on how to define it in different use cases.

The normalized version of this metric, *N*2*DCG*_*u*_ requires defining the *I*2*DCG*_*u*_, which is the 2*DCG*_*u*_ of the *ideal ranking*. In a single list setting the ideal ranking is the list which contains the relevant items in decreasing relevance from the beginning of the list. In the generalized two-dimensional layout, it contains the user's most relevant items, ranked according to decreasing relevance in positions with decreasing position discount. Note that, as done for IDCG, if a user has several relevant items that exceed the recommendation list length *l*, only the *l* most relevant ones will be used to compute the *I*2*DCG*_*u*_. The ideal ranking meets the following constraints: *g*_*ujk*_ ≥ *g*_*uxy*_ if *d*_*jk*_ < *d*_*zx*_ for any pair of items in positions (*j, k*) and (*x, y*).

Finally, *N2DCG* is computed as the average of all *N*2*DCG*_*u*_ as in the traditional NDCG.

### 5.1. Relevance

**Figure 4:**
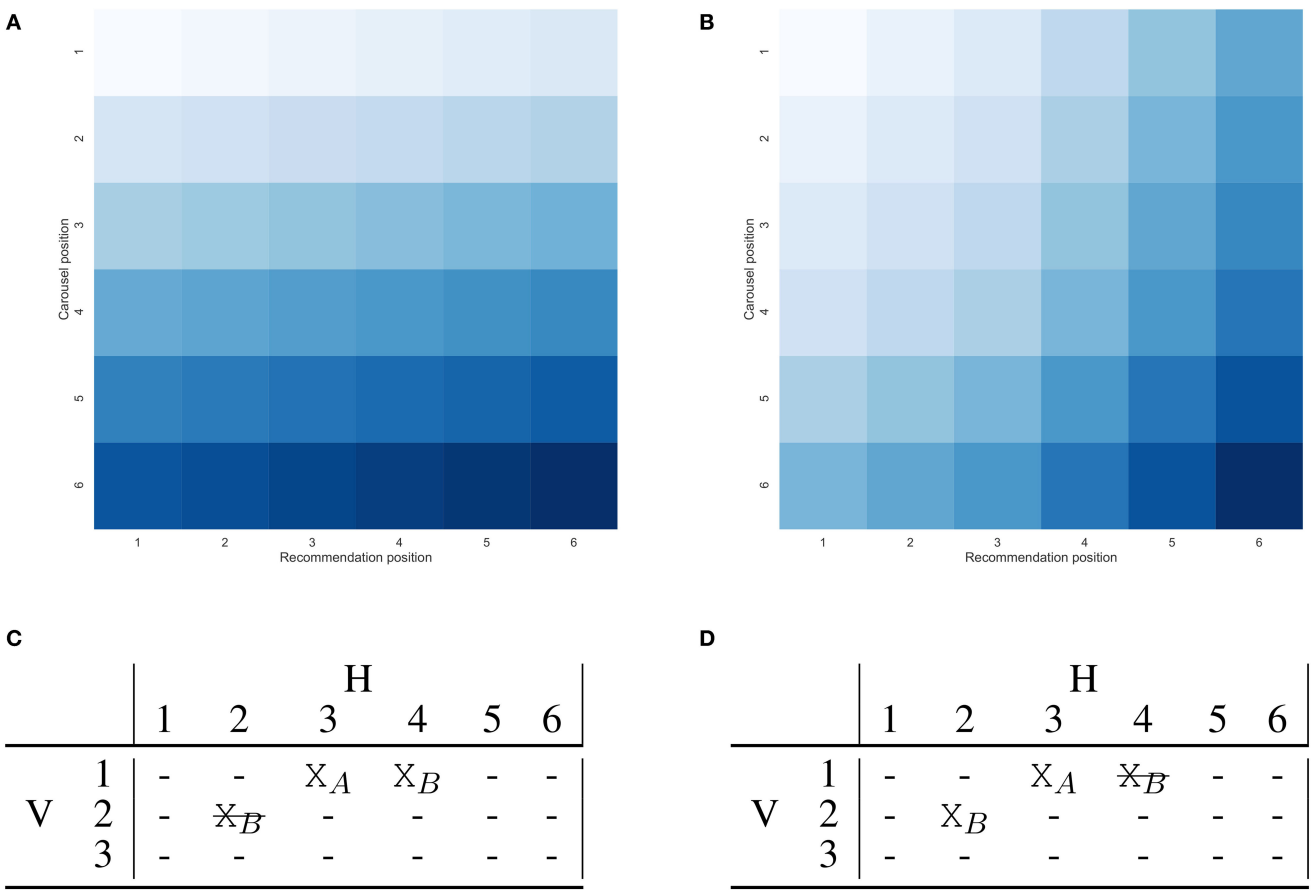
A visual comparison of the two-dimensional discount function under different assumptions: **(A)** Represents carousels concatenated in a single list. **(B)** Represents two-dimensional discount which accounts for the *golden triangle* and number of user actions. **(C,D)** Show the impact of the discount function on the relevance of a correct recommendation. Consider a small user interface with 3 carousels, each with 6 elements. Correct recommendations are represented with “X_*n*_” with *n* the item identifier, while incorrect ones by “-”. Item *B* is recommended correctly both by the first and second recommendation list. Only one of the two will be considered relevant, depending on the discount, the other is shown as crossed out. The single list discount shown in **(A)** will lead to consider relevant the recommendation of item *B* in the *first* carousel, while the two-dimensional discount shown in **(B)** will lead to consider relevant the recommendation in the *second* carousel.

As stated in assumption (2), a relevant item is valuable for the user only when it is first encountered. This means that if a relevant item appears multiple times, each in a different carousel, it should be considered relevant only in its *best* position. We define such position as the one with the lowest *discount*. Function *r*_*ujk*_ should be modified accordingly. Due to this, if an item is correctly recommended twice the position that will be considered relevant may change depending on the discount function applied. An example of this is shown in Figure 4.

### 5.2. Single List Discount

It is possible to represent with N2DCG the scenario where all carousels are concatenated into a single list by calculating the position of the cell in coordinates *j, k* if all carousels lists would be concatenated:

(6) $$\begin{array}{r} d_{jk}^{s}=\frac{1}{\log_{2}\left(\left(j-1\right)H+k+1\right)} \end{array}$$

As previously mentioned, this formulation is not grounded in a realistic scenario because it does not reflect the user behavior (refer to Figure 4A), therefore, we argue that it should not be applied.

### 5.3. Golden Triangle Discount

In order to account for the *golden triangle* behavior, as per assumption (3), the position discount should decrease as the distance of the cell from the top-right corner increases:

(7) $$\begin{array}{r} d_{jk}^{t}=\frac{1}{\log_{2}\left(\alpha j+\beta k\right)} \end{array}$$

The coefficients α and β are two weights that can be used to account for different types of user behaviors. For instance, let us assume a scenario where users are more inclined to explore the vertical dimension. In this case, α should be set to a low value in order to penalize less the vertical dimension. In order to make the discount start from 1, α and β should be ≥ 1 since the base of the logarithm used is 2. Notice that this is true only because we are extending a logarithmic discount function. For other discount functions (Kanoulas and Aslam, 2009; Zhou et al., 2014), the constraints can change.

### 5.4. User Actions Discount

**Figure 5:**
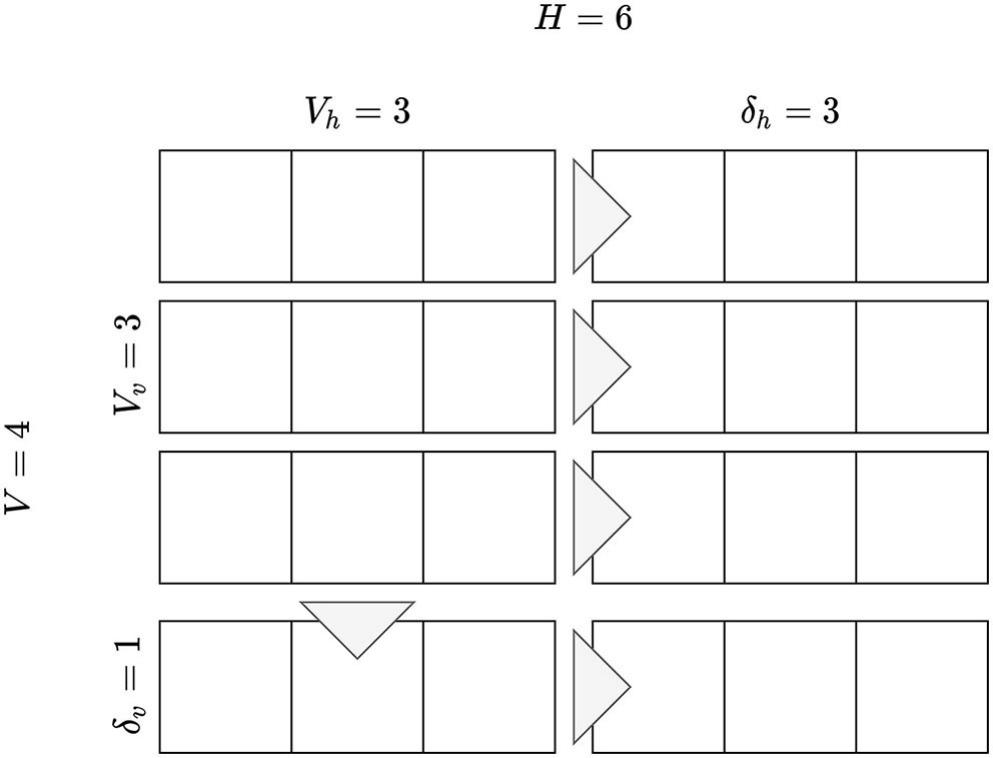
An example interface with 4 carousels of 6 elements each, where 3 carousels, with 3 items each, are visible. A horizontal swipe reveals 3 items, while a vertical swipe reveals one additional carousel.

In order to account for assumption (4), the position discount should decrease the more actions are required by the user to make that position visible. In a carousel interface, there is an initial rectangular portion of the recommendations that are immediately shown to the user. We refer to the number of items visible as *V*_*h*_ (number of visible horizontal items) and the number of carousels visible as *V*_*v*_ (number of visible vertical items), refer to Figure 5. In order to reveal more items in either the horizontal or vertical directions, the user needs to perform a certain action, i.e., scroll the mouse wheel, swipe on mobile devices). We call these actions as *swipe actions*. Each of these actions will reveal a certain number of new items available in the recommendation lists. The number of items revealed with a swipe action is defined as δ_*h*_ ∈ {1, 2, …, *V*_*h*_} (for horizontal swipe actions) and δ_*v*_ ∈ {1, 2, …, *V*_*v*_} (for vertical swipe actions).

For example, on Netflix, every horizontal swipe on a carousel replaces all the items displayed in the carousel, therefore, δ_*h*_ = *V*_*h*_.

The number of horizontal and vertical actions needed to visualize an item in position (*j, k*) (the *k*-th item in carousel *j*) is computed as

(8) $$\begin{array}{l} n_{j}^{\text{h}-\text{swipes}}=\{\begin{array}{ll} \left\lceil \frac{j-V_{h}}{\delta_{h}}\right\rceil & \text{if }j>V_{h}\\ 0 & \text{otherwise} \end{array} \end{array}$$

(9) $$\begin{array}{l} n_{k}^{\text{v}-\text{swipes}}=\{\begin{array}{ll} \left\lceil \frac{k-V_{v}}{\delta_{v}}\right\rceil & \text{if }k>V_{v}\\ 0 & \text{otherwise} \end{array} \end{array}$$

The final discount accounts for both the triangle discount, as defined in (7), and the number of horizontal and vertical user actions required to discover the item

(10) $$\begin{array}{r} d_{jk}^{a}=\frac{1}{\log_{2}\left(\alpha j+\beta k+\gamma n_{j}^{\text{h}-\text{swipes}}+\lambda n_{k}^{\text{v}-\text{swipes}}\right)} \end{array}$$

Notice that this formulation accounts for both vertical and horizontal swipes. The coefficients α, β, γ, λ are four positive weights that can be used to account for different types of user behaviors. The first two weights (α and β) control the general penalization of the vertical and horizontal dimensions, respectively and in order for the total discount to start from 1 α, β ≥ 1. Penalizing more or less the user actions needed to reveal a certain item is possible by controlling γ, λ ≥ 0. For example, it could be that items presented together in the same carousel have a similar probability of interaction (refer to the first 10 elements of the first carousel in Figure 3). Hence, the horizontal dimension should be penalized less. Another possibility is that, on a desktop device, the horizontal swipe done with a mouse click will have a higher weight than the same swipe done with a touch on a mobile device.

For illustrative purposes, let us consider a possible scenario for a mobile device, where the screen contains 4 carousels and 3 recommendations each. We set the horizontal and vertical steps to 1, α, β, γ, λ are set to 1 as well. The resulting discount is shown in Figure 4B.

**Figure 6:**
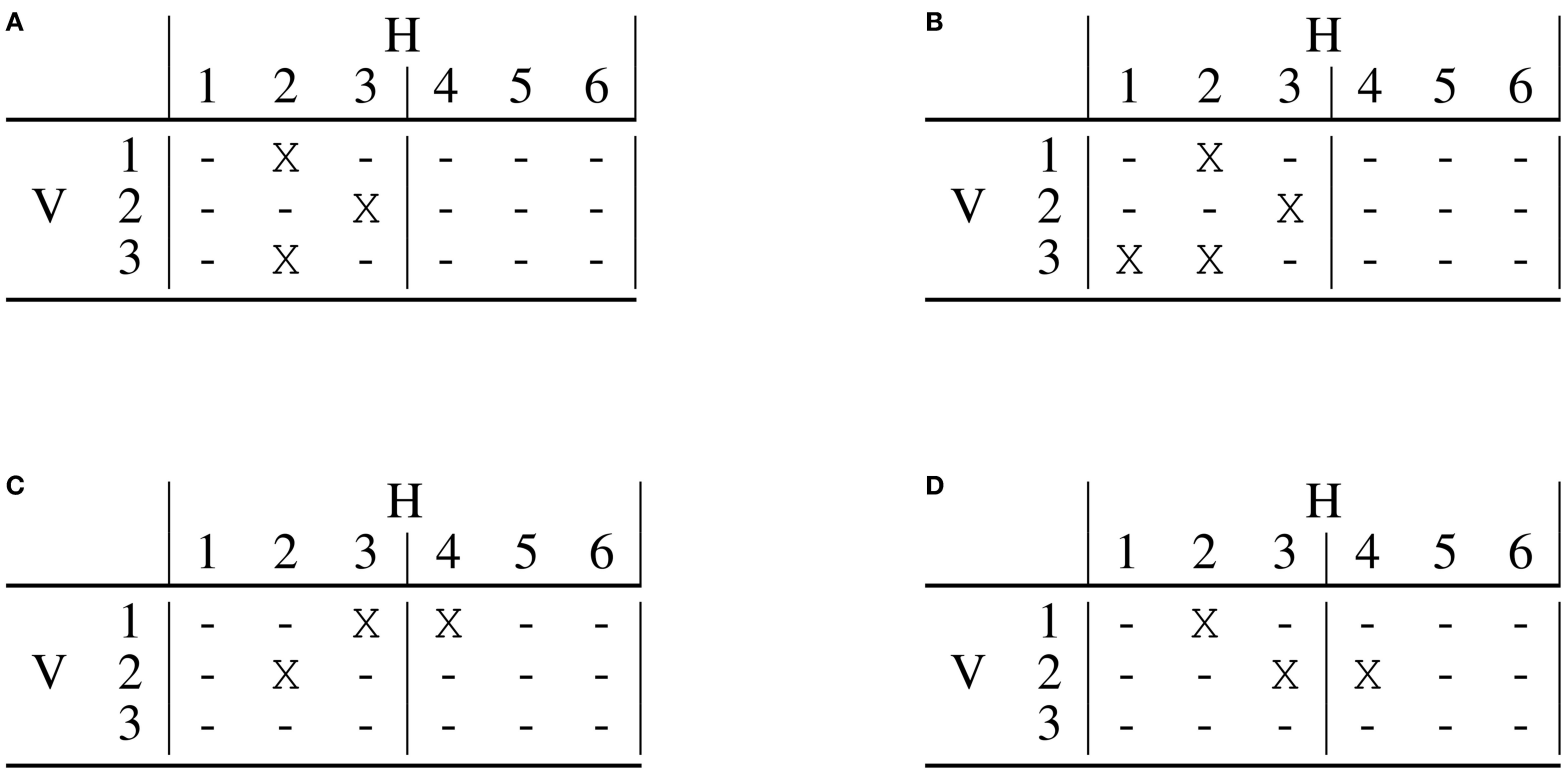
A small user interface with 3 carousels, each with 6 elements on which the behavior of DCG and 2DCG is compared. Correct recommendations are represented with “X” while incorrect ones by “-”.”. The vertical line between columns 3 and 4 represents the limit of the area initially visible to the user, i.e., *V*_*h*_ = 3. **(A,B)** Represent the same layout in which **(B)** contains an additional correct recommendation in position (3, 1). **(C,D)** Represent two different layouts created based on the same three recommendation lists, with DCG preferring layout **(C)** and 2DCG preferring layout **(D)** due to the effect of the user action penalty on the correct recommendation in column 4.

Figure 6 compares the single list DCG and 2DCG in two different scenarios. The user interface has *V* = 3 carousels each of length *H* = 6, with *V* = *V*_*v*_ = 3, *V*_*h*_ = 3, δ_*h*_ = 3 and the penalty for the horizontal user action is γ = 10. The first scenario compares the values when three correct recommendations exist, see Figure 6A, to which a fourth correct recommendation is added in position (3, 1), refer to Figure 6B. The value of DCG increases by 0.263 (from 1.057 to 1.320), while 2DCG increases by 0.497 (from 1.364 to 1.861) indicating how 2DCG is much more affected by the additional correct recommendation than DCG. This is because DCG applies a much higher discount due to the position compared to 2DCG. The effect will become more marked for longer carousel interfaces, making it much more difficult for DCG to discriminate reliably between layouts that differ in their last recommendation lists. For a correct recommendation in position (10, 1), DCG applies a discount 40% higher than that of 2DCG, while only 20% higher if a correct recommendation is in position (30, 1). This means that although the most marked differences are in the first carousels, despite the logarithmic nature of the discount, 2DCG is still able to better account for the user behavior even in long carousel interfaces. Consider that at the time of writing, the Netflix homepage shows more than 30 carousels. The second scenario considers the impact of the user action penalty on determining the optimal layout when three recommendation lists are available. The recommendation lists have zero, one, and two correct recommendations, respectively. In the list with two correct recommendations, the second one is outside the visible area and requires a user action to be seen. The layout depicted in Figure 6C puts first the list with two correct recommendations, then the list with one, and finally the list with zero. This corresponds to a DCG of 1.256 and 2DCG of 1.246. A different layout, obtained by swapping the first two lists as depicted in Figure 6D, has instead a DCG of 1.221 and a 2DCG of 1.312. According to the single list DCG, it is preferable to put the recommendation list with two correct recommendations at the top, while the 2DCG will account for the fact that the second correct recommendation will be outside the user visible area and will have a much lower contribution. Due to this, DCG and 2DCG will lead to the selection of different layouts. This effect will be more or less marked according to the impact of the user action penalty, which will depend on the scenario of interest.

## 6. Experimental Protocol

This section describes the experimental protocol that is used for the analysis reported in Section 7.

### 6.1. Recommendation Lists

In a realistic carousel scenario, several recommendation lists (or carousels) are available generated with different algorithms or editorial rules. In order to mimic this setting, we include in the evaluation 16 algorithms developed in the last three decades of research that are simple, well-known, and competitive (Ferrari Dacrema et al., 2021). The algorithms can be grouped into 6 categories:

- **Non-personalized:** TopPopular, which recommends the most popular items, and GlobalEffects which recommends the highest rated items.
- **Item-based heuristic:** ItemKNN and UserKNN (Sarwar et al., 2001) compute the item-item or user-user similarity based on past user interactions. Both models use cosine similarity with shrinkage to reduce the similarity of items or users with low support.
- **Item-based machine learning:** SLIM (Ning and Karypis, 2011), SLIM BPR, and EASE^*R*^ (Steck, 2019) all compute an item-item similarity by optimizing different criteria. EASE^*R*^ in particular is a recently proposed shallow autoencoder with a closed form solution.
- **Graph-based:** P^3^α (Cooper et al., 2014) and RP^3^β (Paudel et al., 2017) are both simple methods that represent the user interactions as a bipartite user-item graph and model the recommendations by simulating a random walk.
- **Matrix factorization:** PureSVD (Cremonesi et al., 2010), FunkSVD (Ferrari Dacrema et al., 2021), Non-negative matrix factorization (Cichocki and Phan, 2009), MF BPR (Rendle et al., 2009), and IALS (Hu et al., 2008) are all widely known models that represent the user interactions as the product of lower dimensionality matrices learn by optimizing different losses.
- **Content-based and hybrid:** ItemKNN CBF builds an item-item similarity using the item features, while ItemKNN CF-CBF uses both item ratings and item features concatenated in a single vector (Mobasher et al., 2003). Both use cosine with shrinkage as the collaborative ItemKNN.

### 6.2. Hyperparameter Optimization

While this article does not aim to show that any particular model or carousel ranking strategy is superior to others, we nonetheless ensure that all algorithms are fairly and consistently optimized. To do so, we followed the best practices highlighted by Ferrari Dacrema et al. (2021), and we relied on the framework published. The data is split into 80% training, 10% validation, and 10% test with a user-wise random holdout (Antenucci et al., 2018; Ferrari Dacrema et al., 2021)^1^ optimizing the recommendation quality on the validation split, measured with NDCG at cutoff 10. Note that in this case, each model is optimized in a traditional scenario where only a single recommendation list is provided, therefore, there is no need (and indeed at this stage is not possible) to account for the whole user interface. We explored 50 cases for each algorithm, the first 16 used as random initialization. We constrain the optimization to a maximum of 14 days for each recommender model and a maximum of 64GB of RAM. Once the optimization terminates, the final model is fitted on the union of the training and validation data using the best hyperparameters found and is evaluated on the test data. The hyperparameter ranges and distribution used during the optimization are the same used and described in Ferrari Dacrema et al. (2021). The optimal hyperparameters for all recommendation models are reported as part of the Tables A1–A3 in Appendix.

### 6.3. Datasets

We report the results for some widely known and used datasets, all of which are publicly available. We only selected datasets collected from domains that typically adopt a carousel-based user interface, i.e., video-on-demand and music streaming:

- *MovieLens 20M*^2^ (Harper and Konstan, 2016), a popular dataset of movie recommendations. The dataset contains user provided tags for items as well as the year of release and the genre. User ratings are available in a range of 1-5. The dataset has 20.0 M interactions, 27 k items, 138 k users, and a density of 5.3 · 10^−3^.
- *Netflix Prize*^3^ (Bennett and Lanning, 2007), is the well known movie dataset from the *Netflix Prize*. User ratings are available in a range of 1-5. The dataset has 100.4 M interactions, 17 k items, 479 k users, and a density of 1.2 · 10^−2^.
- *ContentWise Impressions* (Pérez Maurera et al., 2020) is a dataset collected from a video-on-demand media provider that contains both user interactions and impressions. The interactions are implicit. The dataset has 4.5 M interactions, 145 k items, 41 k users, and a density of 7.4 · 10^−4^.

### 6.4. Carousel Layouts Heuristics

While the purpose of this article is not to propose a specific algorithm to find an optimal carousel ranking, we believe it is useful to describe two simple heuristic strategies that can be used as baselines to compare with more sophisticated strategies. As previously observed, there are only few studies that deal with the problem of recommendation list selection for a carousel interface. Usually, those studies make assumptions on the recommendation models, leverage specific types of data such as session and context or require an online setting. In other cases, the selection of carousels is part of the recommendation model itself, which means it is not applicable in the scenario where the recommendation models are black boxes. Note that one may choose a single global layout, but also select different layouts for different users or groups of users.

Let's first consider the solution space for the task of carousel selection and ranking:

- **Exhaustive selection with default ranking**: Given a set of available recommendation lists, this baseline evaluates all possible subsets of the desired length, i.e., the number of carousels, with a default ordering that may be selected heuristically. For example, we may wish to select one among many possible carousels related to sports, then another related to a genre, and finally another personalized list. An exhaustive search is very computationally expensive as it corresponds to selecting the combinations without repetitions of *V* carousels within a pool of *M* lists, resulting in a total number of cases $\frac{M!}{V!\left(M-V\right)!}$. This makes an exhaustive search impossible for all but the smallest cases, where the number of available lists is very limited, hence it is of little practical use.
- **Exhaustive selection and ranking**: Given a set of available recommendation lists, the goal is not only to decide which to select, as in the *Exhaustive selection with default ranking* case, but also to decide how to rank them as carousels. This search corresponds to exploring all possible permutations of the selected *V* carousels, which requires evaluating *V*! layouts for each selection, making it again too computationally expensive for practical use.

Two simple greedy strategies that can be used for the selection and ranking of carousels are the following:

- **Individual greedy**: The recommendation lists are selected according to their recommendation quality measured individually on the validation data, and ranked with decreasing values. Therefore, the list with the best recommendation quality will become the first carousel, the second-best will become the second carousel, and so on until the desired number of carousels is reached. In our experiments, we have used NDCG with a cutoff of 10 to rank carousels with the Individual Greedy strategy. This approach cannot account for duplicate recommendations and may select a set of lists with similar recommendations. For example, the second-best list may be very similar to the first one, so most of its correct recommendations will already be present in the first carousel. This strategy requires only the recommendation quality on the validation data which is either already available after the recommendation models that generated the lists have been optimized or can be easily computed once a new editorially curated list is added to the available ones. Hence, the Individual Greedy selection baseline has a low computational cost.
- **Incremental greedy**: This baseline does not select the recommendation lists based on a fixed accuracy value but rather iteratively evaluates all of them, accounting for those that have already been selected. If a recommendation list has high accuracy but provides recommendations similar to those already selected, it will exhibit lower recommendation quality. This carousel selection baseline is better suited to account for the characteristics of a carousel interface, but it is much more computationally expensive requiring to run $\overset{V}{\underset{i=1}{\sum }}M-i+1$ evaluations.

## 7. Results

In this section, we apply the proposed carousel evaluation on numerous widely used algorithms and compare the results obtained with the traditional evaluation which considers each model independently, we also compare the results obtained by optimizing a carousel layout with the traditional single-list NDCG and with the proposed N2DCG. We discuss the results of this comparison and highlight some common trends.

The total number of available recommendation models, i.e., *M*, differs according to the dataset: Movielens 20M has 16 because it includes 14 collaborative and 2 content based ones, Netflix Prize has 14 which corresponds to all the collaborative models, ContentWise Impressions has 12 because SLIM BPR and EASE^*R*^ required more than the available 64GB of RAM and could not be optimized. N2DCG uses the following parameters: *V*_*v*_ = *min*(*V*, 3), *V*_*h*_ = 3, δ_*v*_ = 1, δ_*h*_ = 3, α = β = γ = λ = 1, refer to Figure 4.

### 7.1. Recommendation Quality Under a Carousel Evaluation

The first analysis shows how the relative recommendation quality of a model changes by using a carousel evaluation. In this experiment, the goal is to choose which model to add as the *last* carousel in an interface that contains an increasing number of carousels: TopPopular, ItemKNN CF, and, for the Movielens 20M dataset, ItemKNN CBF.

**Figure 7:**
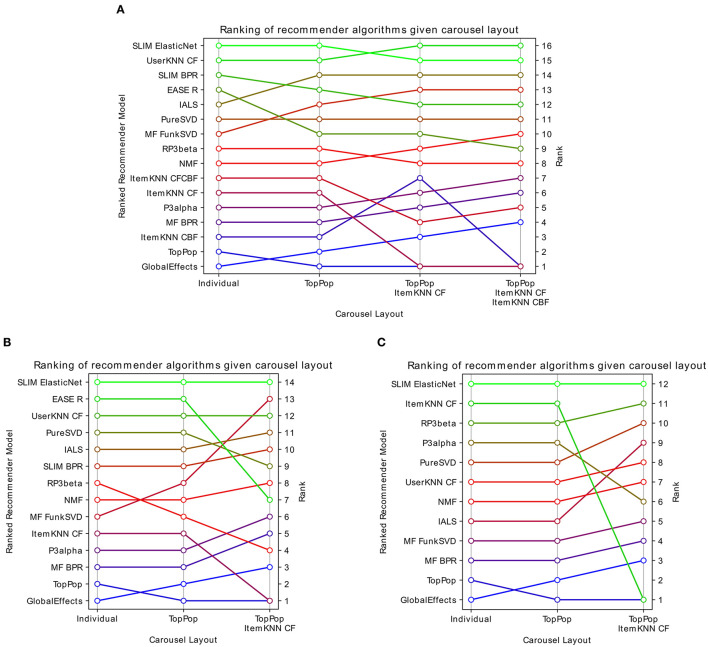
Visualization of how the ranking of several recommendation models changes when they are evaluated independently or as the last recommendation list in a carousel interface of increasing complexity. **(A)** Movielens 20M, **(B)** Netflix Prize, and **(C)** ContentWise Impressions. Highest ranked models are the best performing according to NDCG at 10.

The models are first evaluated individually with the traditional evaluation protocol and then with the proposed carousel evaluation protocol. All recommendation lists have a length, i.e., *H*, of 10. Note that in the individual evaluation, there will be a single recommendation list, while in the carousel evaluation, there will be more than one, therefore, the absolute values of the NDCG are not comparable, for this reason, the analysis will focus on the ranking of the models. The results are reported both as tables and as figures that highlight the changes in the relative ranking of the modes: Figure 7A and Table 2 (Movielens 20M), Figure 7B and **Table 4** (Netflix Prize), Figure 7C and Table 3 (ContentWise Impressions).

**Table 2:** Comparison of the NDCG at 10 and overall model ranking for Movielens 20M.

			**Carousel layout**					
	**Individual**		**TopPop**		**TopPop**		**TopPop**	
					**ItemKNN CF**		**ItemKNN CF**	
							**ItemKNN CBF**	
	**NDCG**	**Rank**	**NDCG**	**ΔRank**	**NDCG**	**ΔRank**	**NDCG**	**ΔRank**
TopPopular	0.1058	15	0.0953	-	0.1607	-	0.1713	-
ItemKNN CF	0.2216	11	0.1653	0	0.1607	-	0.1713	-
ItemKNN CBF	0.1202	14	0.1271	0	0.1734	3	0.1713	-
GlobalEffects	0.0478	16	0.1041	0	0.1659	0	0.1747	0
UserKNN CF	0.3088	2	0.1917	0	0.1958	1	0.1978	1
P^3^α	0.1968	12	0.1393	0	0.1695	0	0.1778	1
RP^3^β	0.2422	8	0.1667	0	0.1743	-1	0.1812	-1
IALS	0.2702	5	0.1868	2	0.1932	2	0.1961	2
MF BPR	0.1645	13	0.1352	0	0.1689	0	0.1776	1
MF FunkSVD	0.2519	7	0.1790	2	0.1922	3	0.1952	3
PureSVD	0.2657	6	0.1765	0	0.1832	0	0.1872	0
NMF	0.2288	9	0.1662	0	0.1819	1	0.1871	2
EASE^*R*^	0.2740	4	0.1756	-3	0.1823	-3	0.1861	-4
SLIM ElasticNet	0.3109	1	0.1920	0	0.1951	-1	0.1964	-1
SLIM BPR	0.2792	3	0.1811	-1	0.1882	-2	0.1918	-2
ItemKNN CFCBF	0.2264	10	0.1662	0	0.1667	-3	0.1759	-2

*Each model is evaluated both individually (single-carousel) and as the last recommendation list in a multi-carousel interface of increasing complexity. The NDCG is computed with the single-list discount (concatenating all carousel lists). Higher ranks indicate better recommendation quality. The rank of models that are already used as carousels is removed. ΔRank is the difference between the rank when evaluated individually and the rank when evaluated in the corresponding carousel layout, e.g., a negative ΔRank indicates the model is in a worse ranking position*.

**Table 3:** Comparison of the NDCG at 10 value and overall model ranking for ContentWise Impressions.

			**Carousel layout**			
	**Individual**		**TopPop**		**TopPop**	
					**ItemKNN CF**	
	**NDCG**	**Rank**	**NDCG**	**Δ Rank**	**NDCG**	**Δ Rank**
TopPopular	0.0708	11	0.0617	-	0.2448	-
ItemKNN CF	0.5328	2	0.2557	0	0.2448	-
GlobalEffects	0.0000	12	0.0617	0	0.2448	0
UserKNN CF	0.3667	6	0.1915	0	0.2688	0
P^3^α	0.4183	4	0.2095	0	0.2686	-4
RP^3^β	0.5016	3	0.2479	0	0.2745	0
IALS	0.3369	8	0.1754	0	0.2696	3
MF BPR	0.1584	10	0.1118	0	0.2554	0
MF FunkSVD	0.2324	9	0.1395	0	0.2624	0
PureSVD	0.3699	5	0.1935	0	0.2712	1
NMF	0.3428	7	0.1798	0	0.2686	0
SLIM ElasticNet	0.5548	1	0.2651	0	0.2769	0

*Each model is evaluated both individually (single-carousel) and as the last recommendation list in a multi-carousel interface of increasing complexity. The NDCG is computed with the single-list discount (concatenating all carousel lists). Higher ranks indicate better recommendation quality. The rank of models that are already used as carousels is removed. ΔRank is the difference between the rank when evaluated individually and the rank when evaluated in the corresponding carousel layout, e.g., a negative ΔRank indicates the model is in a worse ranking position*.

By looking at Figure 7A (Movielens 20M) and Table 2, we can make several considerations. Under the traditional evaluation in which an algorithm is evaluated independently (i.e., individual), we can see that almost all personalized algorithms have a recommendation quality between two and three times better than TopPopular. The only model that is worse than TopPopular is GlobalEffects and the best performing algorithms are SLIM, UserKNN, and EASE^*R*^. If we look at the carousel evaluation, we can see that no algorithm has a lower recommendation quality than TopPopular, which is expected since TopPopular is the predefined first carousel while each algorithm is the second one and therefore the final recommendation quality can only be greater or equal. As a general trend, we can see that the relative effectiveness of the models differs, resulting in changes to the ranking of the algorithms in the two evaluation modes. Some models such as GlobalEffects and PureSVD are always ranked in the same position. Others, in this case, all other matrix factorization algorithms, gain 2 or 3 positions. On the other hand, item-based machine learning models tend to consistently lose some positions, with EASE^*R*^ being the worst affected and losing 4 positions. As a result, while in the individual evaluation the best algorithms are SLIM ElasticNet, UserKNN CF, SLIM BPR, and EASE^*R*^, in the carousel evaluation the best algorithms are UserKNN CF, SLIM ElasticNet, IALS, and FunkSVD. Since the recommendation lists generated by all algorithms are identical for both evaluation procedures, the difference in the ranking lies in how those recommendations intersect. Algorithms that will tend to recommend popular items will be penalized in this carousel evaluation because popular items will already be present in the TopPopular carousel, whereas algorithms providing accurate recommendations but involving less popular items will be advantaged. Similarly, since the second carousel layout adds an ItemKNN CF, the algorithms providing similar recommendations to it will also be penalized.

Figure 7B (Netflix Prize) and Table 4 show a similar, albeit more marked, behavior. The model's ranking is mostly unchanged when the recommendation model is evaluated in a carousel layout having only TopPopular as the first carousels, the only changes are RP^3^β and FunkSVD swapping relative positions. The inclusion of the ItemKNN CF instead causes some sharp changes in ranking, in particular, EASE^*R*^ falls by 6 positions while FunkSVD gains 7. Finally, Figure 7C (ContentWise Impressions) and Table 3 show a case where the ranking is less affected. Using a TopPopular as the first carousel does not change the relative ranking of the models. Including an ItemKNN CF as the second carousel affects mostly two models, P^3^α which loses 4 positions, and IALS which gains 3.

**Table 4:** Comparison of the NDCG at 10 value and overall model ranking for NetflixPrize.

			**Carousel layout**			
	**Individual**		**TopPop**		**TopPop**	
					**ItemKNN CF**	
	**NDCG**	**Rank**	**NDCG**	**Δ Rank**	**NDCG**	**Δ Rank**
TopPopular	0.0799	13	0.0678	-	0.1261	-
ItemKNN CF	0.2060	10	0.1335	0	0.1261	-
GlobalEffects	0.0159	14	0.0733	0	0.1282	0
UserKNN CF	0.2581	3	0.1516	0	0.1573	0
P^3^α	0.1810	11	0.1194	0	0.1478	1
RP^3^β	0.2209	7	0.1345	-2	0.1422	-4
IALS	0.2380	5	0.1497	0	0.1566	1
MF BPR	0.1656	12	0.1172	0	0.1426	1
MF FunkSVD	0.2077	9	0.1446	2	0.1639	7
PureSVD	0.2508	4	0.1515	0	0.1552	-2
NMF	0.2192	8	0.1434	0	0.1546	1
EASE^*R*^	0.2619	2	0.1520	0	0.1534	-6
SLIM ElasticNet	0.2913	1	0.1662	0	0.1669	0
SLIM BPR	0.2353	6	0.1467	0	0.1561	1

*Each model is evaluated both individually (single-carousel) and as the last recommendation list in a multi-carousel interface of increasing complexity. The NDCG is computed with the single-list discount (concatenating all carousel lists). Higher ranks indicate better recommendation quality. The rank of models that are already used as carousels is removed. ΔRank is the difference between the rank when evaluated individually and the rank when evaluated in the corresponding carousel layout, e.g., a negative ΔRank indicates the model is in a worse ranking position*.

Overall, no clear pattern emerges, with the different recommendation models being affected in different ways according to the carousel structure and the dataset. An observation that can be made is, e.g., the consistent drop in positions of the item-based machine learning models in Movielens 20M indicating that the *correct* recommendations they provide are similar to those of the already available carousels, in particular, the ItemKNN ones. This behavior depends on the dataset, with the models most negatively affected by the presence of an ItemKNN carousel being P^3^α on ContentWise Impressions and EASE^*R*^ on the Netflix Prize dataset.

These results indicate the importance of accounting for how a set of recommendation lists complement each other and that this effect can change substantially the relative ranking of some algorithms compared to when they are evaluated individually, therefore, leading to different conclusions on which is the best recommendation list to include.

### 7.2. Selecting a Carousel Layout

Table 5 compares the recommendation quality of the carousel layout selected with different approaches on interfaces with an increasing number of carousels, from 2 to 8. The recommendation quality is measured with, and optimized for, NDCG at 10. The same experiment was also conducted optimizing N2DCG and produced consistent results. Although the maximum number of carousels reported here is 8, note that some content providers use much longer user interfaces, with Netflix showing more than 30 carousels.

**Table 5:** Comparison of the NDCG at 10 of the layout selected according to the two proposed greedy strategies and compared to the exhaustive search of all possible model selections and rankings.

		**Number of carousels**						
		**2**	**3**	**4**	**5**	**6**	**7**	**8**
**Movielens 20M (***M* = 16**)**								
Solution space size	Number of rankings	2.4 · 10^2^	3.3 · 10^3^	4.3 · 10^4^	5.2 · 10^5^	5.7 · 10^6^	5.7 · 10^7^	5.1 · 10^8^
	Number of selections	1.2 · 10^2^	5.6 · 10^2^	1.8 · 10^3^	4.3 · 10^3^	8.0 · 10^3^	1.1 · 10^4^	1.2 · 10^4^
Selection method	Exhaustive selection and ranking	0.3168	0.3220	-	-	-	-	-
	Exhaustive selection default ranking	0.3168	0.3220	0.3266	0.3296	-	-	-
	Incremental greedy	0.3168	0.3220	0.3266	0.3297	0.3320	0.3345	0.3363
	Individual greedy	0.3168	0.3180	0.3190	0.3241	0.3250	0.3295	0.3309
**Netflix Prize (***M* = 14**)**								
Solution space size	Number of rankings	1.8 · 10^2^	2.1 · 10^3^	2.4 · 10^4^	2.4 · 10^5^	2.1 · 10^6^	1.7 · 10^7^	1.2 · 10^8^
	Number of selections	9.1 · 10^1^	3.6 · 10^2^	1.0 · 10^3^	2.0 · 10^3^	3.0 · 10^3^	3.4 · 10^3^	3.0 · 10^3^
Selection method	Exhaustive selection and ranking	0.2781	-	-	-	-	-	-
	Exhaustive selection default ranking	0.2781	0.2809	-	-	-	-	-
	Incremental greedy	0.2781	0.2812	0.2830	0.2865	0.2891	0.2912	0.2931
	Individual greedy	0.2692	0.2701	0.2689	0.2714	0.2740	0.2761	0.2777
**ContentWise impressions (***M* = 12**)**								
Solution space size	Number of rankings	1.3 · 10^2^	1.3 · 10^3^	1.1 · 10^4^	9.5 · 10^4^	6.6 · 10^5^	3.9 · 10^6^	2.0 · 10^7^
	Number of selections	6.6 · 10^1^	2.2 · 10^2^	4.9 · 10^2^	7.9 · 10^2^	9.2 · 10^2^	7.9 · 10^2^	4.9 · 10^2^
Selection method	Exhaustive selection and ranking	0.5162	0.5099	0.5133	0.5153	-	-	-
	Exhaustive selection default ranking	0.5162	0.5099	0.5132	0.5152	0.5178	0.5199	0.5213
	Incremental greedy	0.5162	0.5099	0.5133	0.5153	0.5179	0.5201	0.5215
	Individual greedy	0.5162	0.5098	0.5123	0.5149	0.5172	0.5183	0.5190

*The page layout contains from 2 to 8 carousels. Results for exhaustive searches requiring more than a week of computation are missing*.

As a general observation, we can see how the solution space grows markedly and becomes unpractical to explore exhaustively even for rather small interfaces. For example, on Movielens 20M an interface of 4 carousels corresponds to 1.8 · 10^3^ possible selections and 4.3 · 10^4^ possible rankings. We limit the analysis to the exhaustive searches that could complete in a week of computation. Note that depending on the dataset this corresponds to very different numbers of layouts, from a minimum of 3.6 · 10^2^ for Netflix Prize to a maximum of 9.4 · 10^4^ for ContentWise Impressions. In this experiment, the default ranking adopted by the Exhaustive Selection method is to order the models according to their decreasing recommendation quality when evaluated individually.

Overall, the results indicate that the Exhaustive Selection achieves almost identical overall recommendation quality when compared to the Exhaustive Permutation in the limited number of cases where it is possible to use it. Indeed a difference may emerge for a higher number of carousels but the growth of the search space makes such an analysis impractical. A more interesting analysis can be done with the proposed two greedy strategies. The Incremental Greedy method provides better results than the Individual Greedy, indicating again the importance of accounting for how the recommendation list complement each other. This difference is more marked on denser datasets, i.e., Netflix Prize, and for longer carousel layouts. The only dataset in which it is possible to apply the Exhaustive Selection up to 8 carousels is ContentWise Impressions, where we can see how the Incremental Greedy selects a layout of even better overall recommendation quality than the Exhaustive Selection. This should not be surprising since the exhaustive search strategies find the global optima, which makes them more prone to overfitting and hence exhibiting reduced generalizability. These results indicate that, although very simple, the Incremental Greedy strategy we described is indeed effective to account for how the different recommendation lists complement each other.

### 7.3. Comparing NDCG and N2DCG

The different results obtained by optimizing NDCG or N2DCG are not directly comparable in their absolute value, therefore, are best visualized by comparing the corresponding optimal carousel layouts.

Table 6 compares the optimal layouts for 5 carousels obtained by all exhaustive searches and greedy strategies optimizing both NDCG and N2DCG. This is the longest interface for which it is possible to report results for all four strategies. By first comparing the exhaustive search strategies, we can see that in both cases, Exhaustive Search with Ranking selects the same models, but there is always a couple that is swapped compared to the default Ranking strategy, indicating that a better ranking was found compared to the default one (i.e., decreasing order of individual accuracy). When optimizing the traditional single list NDCG both greedy strategies choose the same first two carousels the exhaustive strategies selected, but then the last three become different in both the ranking of the models and which ones are selected, i.e., Incremental Greedy selects P^3^α while Individual Greedy does not and instead selects PureSVD, while RP^3^β and UserKNN CF are present in both but for different positions.

**Table 6:** Layouts of 5 carousels selected for the ContentWise Impressions dataset selected according to different strategies and optimizing both the single list NDCG and the proposed two-dimensional N2DCG.

**Optimized** **metric**	**Exhaustive selection** **and ranking**	**Exhaustive selection** **default ranking**	**Incremental** **greedy**	**Individual** **greedy**
NDCG	SLIM ElasticNet	SLIM ElasticNet	SLIM ElasticNet	SLIM ElasticNet
	ItemKNN CF	ItemKNN CF	ItemKNN CF	ItemKNN CF
	UserKNN CF	RP^3^β	RP^3^β	UserKNN CF
	RP^3^β	UserKNN CF	P^3^α	RP^3^β
	FunkSVD	FunkSVD	UserKNN CF	FunkSVD
N2DCG	ItemKNN CF	ItemKNN CF	SLIMElasticNet	-
	P^3^α	RP^3^β	ItemKNN CF	-
	RP^3^β	P^3^α	UserKNN CF	-
	PureSVD	PureSVD	RP^3^β	-
	FunkSVD	FunkSVD	FunkSVD	-

*The Individual Greedy layout for N2DCG is missing since that method is based on the traditional single list evaluation*.

When optimizing the N2DCG the optimal layout is quite different, SLIM ElasticNet, which was consistently in the first position when optimizing NDCG, is not even selected, while PureSVD is selected and put toward the end of the layout. The Incremental Greedy strategy selects the same first two carousels that were selected when optimizing NDCG, but the remaining three include different models with a different rankings. This highlights the intrinsic limitations of the simple Incremental Greedy strategy which, due to its definition, will not be able to account for the two dimensional structure of the interface when the very first carousel is selected and will, therefore, more likely find a suboptimal layout.

Similar observations can be made for the other two datasets and a longer user interface. Table 7 shows the optimal layouts of 8 carousels selected for the Netflix Prize and Movielens 20M. On Movielens 20M, again the Incremental and Individual Greedy strategies are similar only in the first carousels but then result in quite different rankings and selected models, and the same holds for the ranking obtained by optimizing N2DCG. The Netflix dataset is instead an example of the case where the Incremental Greedy is ineffective and selects the same layout by optimizing NDCG or N2DCG, indicating this is a scenario where developing a better strategy will be particularly important.

**Table 7:** Layouts of 8 carousels selected for the Movielens 20M and Netflix Prize datasets selected according to different strategies and optimizing both the single list NDCG and the proposed two-dimensional N2DCG.

**Movielens 20M**			**Netflix prize**		
**NDCG**		**N2DCG**	**NDCG**		**N2DCG**
**Incremental greedy**	**Individual greedy**	**Incremental greedy**	**Incremental greedy**	**Individual greedy**	**Incremental greedy**
UserKNN CF	UserKNN CF	UserKNN CF	SLIM ElasticNet	SLIM ElasticNet	SLIM ElasticNet
SLIM ElasticNet	SLIM ElasticNet	SLIM ElasticNet	FunkSVD	EASE^*R*^	FunkSVD
FunkSVD	SLIM BPR	FunkSVD	UserKNN CF	UserKNN CF	UserKNN CF
IALS	EASE^*R*^	IALS	MF BPR	PureSVD	MF BPR
MF BPR	IALS	MF BPR	IALS	SLIM BPR	IALS
NMF	PureSVD	ItemKNN CBF	P^3^α	IALS	P^3^α
ItemKNN CBF	FunkSVD	NMF	NMF	RP^3^β	NMF
ItemKNN CF	RP^3^β	ItemKNN CF	ItemKNN CF	ItemKNN CF	ItemKNN CF

*The Individual Greedy layout for N2DCG is missing since that method is based on the traditional single list evaluation*.

Overall this indicates that optimizing N2DCG, especially for longer page layouts, is a more difficult and nuanced problem, which opens new directions for future research, e.g., representing it as a quadratic optimization problem as done by Ferrari Dacrema et al. (2021).

## 8. Conclusion

This article proposes a new offline evaluation protocol for a carousel user interface, where the recommendation quality of a model is not measured independently but rather is put into the context of other recommendation lists being already available to the users. The experimental analysis shows that the relative ranking of the personalized algorithms changes when accounting for the presence of other carousels in the interface. This confirms previous observations that the correlations between recommendation lists have an important role to play and should be taken into account during offline evaluation as well. Results also show the impact of accounting for how the users navigate a user interface, with the traditional NDCG and the proposed N2DCG resulting in different optimal layouts when applying both exhaustive and greedy strategies. In future study, online studies should be conducted to measure how closely the offline carousel evaluation is able to represent user behavior. For example, one could estimate appropriate factors to be used by the discount term of the N2DCG according to the layout structure and the user action type or explore different relevance functions that attribute partial relevance to duplicate items as well. Another direction is the development of recommendation models specifically tailored for the carousel scenario, e.g., that target specific items that are not accurately recommended by other algorithms (unpopular items), as well as new efficient strategies to build an optimal carousel layout possibly personalized to communities of users or even single users. Ultimately, the carousel evaluation protocol opens new research directions by allowing researchers to conduct offline evaluations in these industrially relevant scenarios and open a wide number of research possibilities in studying how to combine the strength of various models and techniques to provide the user with ever more accurate and interesting recommendations.

## Data Availability Statement

The original contributions presented in the study are included in the article/supplementary material, further inquiries can be directed to the corresponding author.

## Author Contributions

MF and NF wrote the first draft of the manuscript and performed the experiments. All authors contributed to the conception and design of the study and contributed to manuscript revision, read, and approved the submitted version.

## Conflict of Interest

PC was employed by company ContentWise. The remaining authors declare that the research was conducted in the absence of any commercial or financial relationships that could be construed as a potential conflict of interest.

## Publisher's Note

All claims expressed in this article are solely those of the authors and do not necessarily represent those of their affiliated organizations, or those of the publisher, the editors and the reviewers. Any product that may be evaluated in this article, or claim that may be made by its manufacturer, is not guaranteed or endorsed by the publisher.

## Footnotes

^1^The framework implementation relies on the Scikit-optimize package https://scikit-optimize.github.io/.

^2^ https://grouplens.org/datasets/movielens/20m/

^3^ https://www.kaggle.com/netflix-inc/netflix-prize-data

## Appendix

### Optimal Hyperparameters

This section reports the optimal hyperparameters found for all recommendation models and datasets. The hyperparameter ranges and distribution used during the optimization are the same used and described in Ferrari Dacrema et al. (2021). Table A1 refers to the collaborative KNN models, Table A2 to the content based and hybrid KNN models, and Table A3 to the machine learning and graph based models.

**Table A1:** Hyperparameter values for collaborative KNN recommender algorithms on all datasets.

**Algorithm**	**Hyperparameter**	**ContentWise Impressions**	**Netflix Prize**	**Movielens 20M**
UserKNN CF	topK	297	1000	835
	shrink	102	0	0
	similarity	cosine	cosine	cosine
	normalize	True	True	True
	feature weighting	TF-IDF	none	none
ItemKNN CF	topK	5	72	214
	shrink	0	0	982
	similarity	cosine	cosine	cosine
	normalize	True	True	True
	feature weighting	none	TF-IDF	none

**Table A2:** Hyperparameter values for content based and hybrid KNN recommender algorithms.

**Algorithm**	**Hyperparameter**	**Movielens 20M**
ItemKNN CBF	topK	1000
	shrink	1000
	similarity	cosine
	normalize	True
	feature weighting	TF-IDF
ItemKNN CF CBF	topK	253
	shrink	203
	similarity	cosine
	normalize	True
	feature weighting	BM25
	ICM weight	0.1520

*Only Movielens 20M contains item features, the other datasets are, therefore, omitted*.

**Table A3:** Hyperparameter values for machine learning and graph based recommender algorithms on all datasets.

**Algorithm**	**Hyperparameter**	**ContentWise Impressions**	**Netflix Prize**	**Movielens 20M**
P^3^α	topK	146	5	1000
	alpha	1.2748	0.9150	1.0915
	normalize similarity	True	False	True
RP^3^β	topK	5	173	245
	alpha	0.0000	0.7040	0.5775
	beta	0.3487	0.6578	0.5625
	normalize similarity	True	True	True
SLIM ElasticNet	topK	1000	503	588
	l1 ratio	1.14E-02	1.33E-02	2.17E-01
	alpha	0.0010	0.0010	0.0023
MF BPR	sgd mode	adagrad	adagrad	adagrad
	epochs	1415	1500	1065
	num factors	200	200	158
	batch size	2	1	256
	positive reg	2.32E-04	1.00E-05	1.00E-05
	negative reg	1.50E-04	1.00E-05	7.03E-05
	learning rate	5.86E-02	2.96E-02	2.16E-02
MF FunkSVD	sgd mode	adam	adam	adagrad
	epochs	500	280	160
	use bias	False	False	False
	batch size	32	1024	2
	num factors	184	186	127
	item reg	3.92E-04	5.98E-04	1.47E-05
	user reg	2.84E-03	9.44E-04	3.18E-03
	learning rate	1.62E-04	1.10E-03	7.11E-02
	negative quota	0.0133	0.1198	0.0266
PureSVD	num factors	350	47	29
NMF	num factors	324	79	79
	solver	mult. update	mult. update	mult. update
	init type	nndsvda	random	random
	beta loss	frobenius	frobenius	kullback-leibler
IALS	num factors	200	63	60
	epochs	5	65	60
	confidence scaling	linear	linear	linear
	alpha	0.7942	0.2199	0.7748
	epsilon	0.1012	0.0010	0.1358
	reg	1.00E-02	1.00E-02	1.00E-02
EASE^*R*^	l2 norm	-	2.12E+06	7.52E+05
SLIM BPR	topK	-	36	685
	epochs	-	595	970
	symmetric	-	True	True
	sgd mode	-	sgd	sgd
	lambda i	-	2.07E-03	1.00E-05
	lambda j	-	9.20E-03	1.00E-05
	learning rate	-	9.22E-04	1.56E-03

*Results for EASE^R^ and SLIM BPR are missing from the ContentWise Impressions dataset because the algorithms required more than the available 64GB of RAM*.
